# Modifying the Structural and Functional Properties of Walnut Glutenin Through Atmospheric Cold Plasma Treatment: Evaluation of Treatment Times Effects

**DOI:** 10.3390/foods14132289

**Published:** 2025-06-27

**Authors:** Yanmei Deng, Guohui Yuan, Tongqin Yang, Baoyu Gao, Yanling Lu, Jiaojiao Yang, Lei Guo, Qian Ma, Fangyu Fan

**Affiliations:** 1 College of Biological and Food Engineering, Southwest Forestry University, Kunming 650224, China; 14736649465@163.com (Y.D.); 15894518605@163.com (G.Y.); 18385703790@163.com (T.Y.); gaobaoyu66@163.com (B.G.); luyanling20220222@163.com (Y.L.); aw11p6@163.com (J.Y.); guoleigift.student@sina.com (L.G.); 2 Key Laboratory of Forest Disaster Warning and Control of Yunnan Province, Kunming 650224, China; 3 Forest Resources Exploitation and Utilization Engineering Research Center for Grand Health of Yunnan Provincial Universities, Kunming 650224, China

**Keywords:** atmospheric cold plasma, walnut glutenin, physicochemical properties, secondary and tertiary structural, differential scanning calorimetry

## Abstract

Walnut gluten (WGLU) is a plant-based protein rich in essential amino acids for the human body. Due to its poor water solubility and functional properties, its application in the food industry is limited. For the first time, this study looks into how different durations (0, 30, 60, 90, and 120 s) of atmospheric cold plasma (ACP) treatment affect the structure and functional properties of WGLU. ACP processing destroys the spatial structure of the WGLU and alters its functional properties. The comprehensive performance reached its best after 60 s of ACP treatment, the main manifestations included increased β-sheet content, reduced α-helix content, and unfolding of the tertiary structure, which ultimately improved the stability of emulsification and foam. Meanwhile, the solubility (86.35%), water retention rate (2.15 g/g), oil retention rate (5.31 g/g), emulsification rate (10.59 m^2^/g), and foaming rate (24.67%) of WGLU reached their maximum values. However, longer treatment times (90 and 120 s) induce WGLU aggregation, followed by decreased functional properties. In summary, the physicochemical and functional properties of WGLU can be significantly enhanced through ACP treatment, enhancing the bioavailability of gluten and providing an effective strategy for its application in food processing.

## 1. Introduction

Plant proteins are defined as natural, renewable, and biodegradable functional macromolecules. They commonly replace or supplement animal proteins and plant-based meats, dairy alternatives, protein powders, energy bars, and baked goods [1,2,3]. Compared to other plant proteins, the walnut protein includes eight essential amino acids, especially rich in arginine content, which makes it a high-quality, nutritionally and biologically valuable source of plant protein [4,5]. In recent years, the modification of walnut proteins to improve their functional properties has become a hot research topic. Zhang et al. [6] showed that ultrasonic treatment improved the water solubility, emulsification, and emulsion stability of walnut isolate protein. Feng et al. [7] found that walnut protein was enzymatically digested by papain, and it had better emulsification and antioxidant properties. These studies provide some basis for studying walnut proteins. However, the specific mechanisms affecting the functional properties of walnut proteins still need to be explored in depth. Walnut gluten (WGLU) constitutes the primary part of walnut protein, and an in-depth exploration of its structure and functional properties can not only elucidate the physicochemical properties and biological activities of walnut protein but also provide an important theoretical basis for the high-value utilization of walnut resources and the development of functional foods. However, WGLU is a water-insoluble protein with poor solubility, emulsification, and foaming properties, and much of it ends up as feed or is discarded, causing a considerable waste of walnut protein resources [8].

Atmospheric cold plasma (ACP) treatment, as a novel non-thermal treatment technology, has been widely used to alter protein structure and function. ACP induces protein covalent bond rupture by ionizing the air to produce high-energy reactants, such as reactive oxygen species (ROS) and reactive nitrogen species (RNS), ultraviolet photons, and so on, which can disrupt the three-dimensional structure of proteins. In addition, these ROS and RNS transfer peptide bonds, thereby generating compelling oxygen radical-induced structural unfolding of proteins, which alters their secondary and tertiary structures, and improves their emulsification, solubility, and foaming properties [9]. Recently, emphasis has been placed on operating at low temperatures, being energy efficient, and preserving compounds sensitive to heat. ACP treatment utilizes air as the reaction gas medium. During the discharge process, the temperature of the electrons generated by the high-energy electric field is considerably higher than that of the ions. Consequently, the electrons experience minimal energy loss when colliding with ions or neutral particles, thereby preventing an increase in the air’s temperature [10]. It has been reported to improve sunflower proteins’ emulsification properties by ACP treatment [11]. In addition, prolonged ACP treatment time (1–10 min) leads to changes in the secondary and tertiary structure of soybean isolate proteins (SPI), which improves emulsification capacity and foaming ability [12]. However, there are fewer studies on ACP altering the thermal stability and water solubility of glutenin. Meanwhile, few studies have been reported on the interaction mechanism of ACP treatment on the changes in structural and physicochemical properties of WGLU and the time relationship.

Therefore, this research systematically assessed the different ACP treatment times (0, 30, 60, 90, and 120 s) on the structural and functional attributes of WGLU. Firstly, fluorescence spectroscopy, Fourier transform infrared spectroscopy (FTIR), and X-ray diffraction (XRD) were used to investigate the structural effects of different ACP treatment durations on WGLU. Simultaneously, scanning electron microscopy (SEM) was used to observe the microstructural changes of WGLU under different ACP treatment times. On this basis, the influence of ACP treatment at different times on thermal stability, surface hydrophobicity, solubility, water-holding capacity, foaming characteristics, and emulsification characteristics of WGLU was further investigated. The outcomes of this research offer innovative approaches to change the structure and functional properties of WGLU, as well as laying the groundwork for expanding the use of walnut protein resources into new areas.

## 2. Materials and Methods

### 2.1. Materials

Walnuts were purchased from Yangbi, Dali, Yunnan Province. Food-grade soybean oil was sourced from Yihai Kerry Food Industry Co., Ltd. (Kunming, China). Other chemicals used were of analytical grade.

### 2.2. Preparation of WGLU

**Figure 1:**
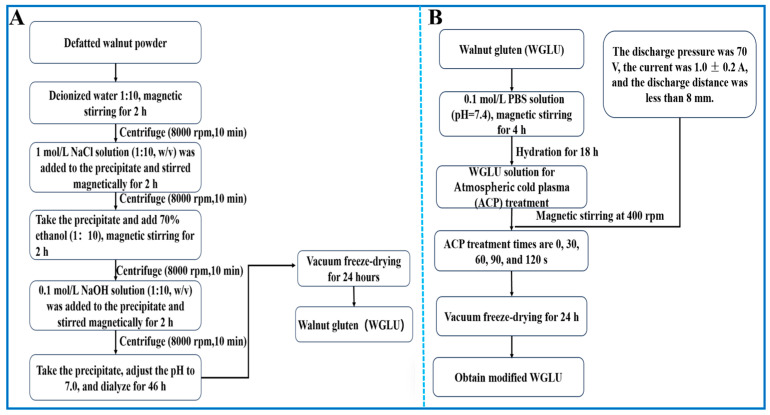
Preparation process of WGLU (**A**) and WGLU treated at different times by ACP (**B**). WGLU, walnut glutenin; ACP, atmospheric cold plasma.

As shown in Figure 1A, WGLU was prepared by first extracting defatted walnut powder sequentially with deionized water, 1 M NaCl, and 70% ethanol in a 1:10 ratio with magnetic stirring for 1.5 h. After each extraction step, centrifuge at 8000 rpm for 10 min and recover the precipitate. Finally, extraction was carried out with 0.1 M NaOH, and the supernatant was taken. The pH was adjusted to 4.5 (1 mol/L HCl), and the precipitate was left to settle for 12 h. The precipitate was centrifuged at 5000 rpm for 20 min, and the precipitate was collected. A total of 50 mL of distilled water was added to the precipitate and stirred magnetically at 500 rpm for 10 min, the pH was adjusted to 7.0 (0.1 mol/L NaOH), it was dialyzed for 36 h, and it was vacuum freeze-dried (48 h) to obtain WGLU [13].

### 2.3. Cold Plasma Treatment of WGLU

As shown in Figure 1B, the WGLU solution, with a concentration of 0.1 g/mL, was made using a 0.1 mol/L phosphate buffer solution (PBS) at pH 7.4, and magnetically stirred at 500 rpm for 4 h. After being hydrated for 18 h at 4 °C, 200 mL of WGLU solution was subjected to ACP treatment under magnetic stirring at 400 rpm (CTP-2000K, Nanjing Suman Electronics Company, Ltd., Nanjing, China). The treatment times were set at 30, 60, 90, and 120 s, with a duration of 0 s serving as the control condition. Carrier gas is air, temperature is 25 ± 2 °C, relative humidity is 45–5%, discharge pressure is 70 V, current is 1.0 ± 0.2 A, and discharge distance is less than 8 mm. The WGLU solution was vacuum freeze-dried after ACP treatment [14].

### 2.4. Structural Properties of WGLU

#### 2.4.1. Measurement of Fluorescence Spectroscopy 

A WGLU solution with a 0.01 g/mL concentration was made using a 0.1 mol/L PBS solution at pH 7.4. The fluorescence spectrum of the WGLU was obtained using an enzyme marker (EPOCH2, BioTek Instruments, Inc., Winooski, VT, USA). An excitation wavelength of 280 nm was used, with an emission wavelength range between 300 and 450 nm, and a slit width of 2 nm [13].

#### 2.4.2. Measurement of Fourier Transform Infrared (FTIR) Spectroscopy 

The functional group of the WGLU was analyzed using an infrared spectrometer (650, Tianjin, China). The WGLU was subjected to 32 scans at a resolution of 4 cm^−1^, spanning wavenumbers from 4000 cm^−1^ to 400 cm^−1^. To assess changes in secondary structure content, Gaussian area fitting was conducted using Peakfitv4.12 [5].

#### 2.4.3. Measurement of X-Ray Diffraction (XRD) 

The crystalline structure was examined using an X-ray diffractometer (Ultima IV, Rigaku Corporation, Akishima, Japan) with a scanning rate of 5°/min and a range from 5° to 90°. The crystallinity was examined using JADE 6.0 software [14].

#### 2.4.4. Measurement of Scanning Electron Microscope (SEM) Analysis

Using a scanning electron microscope (FE-SEM, Sigma 300, Zeiss, Germany) at 15 kV and 10,000× magnification, the microstructures of the WGLU were observed after gold plating [14].

### 2.5. Physicochemical Properties of Walnut Glutenin (WGLU)

#### 2.5.1. Measurement of Particle Size and Zeta Potential

A WGLU solution with a concentration of 0.01 g/mL was made using a 0.1 mol/L PBS solution at pH 7.4, and the size of particles and zeta potential for WGLU were assessed with a nanoparticle size and zeta potential analyzer (Brookhaven Instruments, New York, NY, USA) [5].

#### 2.5.2. Measurement of Total and Active Sulfhydryl (-SH)

A mixture was prepared by adding 1 mL of 5 mg/mL WGLU solution to 5 mL of Tris-glycine buffer (8 M urea, 0.086 mol/L Tris, 0.09 mol/L glycine, 4 mmol/L EDTA, pH 8.0) and 200 μL of Ellman’s reagent. Then, the mixture was reacted for 30 min and centrifuged at 8000 rpm for 10 min. The supernatant was collected and the absorbance at 412 nm was determined. A similar method was used to determine active free thiol groups without adding urea. The total -SH groups and active -SH groups were calculated according to Equation (1) [13].

(1) $$-\mathrm{SH}\;(\mu \mathrm{mol}/g)=\frac{73.53\times A_{412}\times D}{C}$$

where *A*_412_ indicates the absorbance at 412 nm, with *D* as the dilution factor and *C* as the concentration of the WGLU solution (5 mg/mL).

#### 2.5.3. Measurement of Thermal Stability 

WGLU was analyzed by thermal stability using a differential scanning calorimeter (DSC) (3500 Sirius, Netzsch, Germany). The nitrogen flow rate was set at 20 mL/min, the heating rate was set at 10 °C/min, and the heating temperature was set at 20–180 °C [5].

#### 2.5.4. Measurement of Surface Hydrophobicity (H_0_) 

Prepare a WGLU solution at a concentration of 0.1 to 0.5 mg/mL in 0.1 mol/L PBS at pH 7.4. Then, 20 μL of 8 mM 8-(phenylamino)-1-naphthalene sulfonate (ANS) was added to 4 mL of WGLU solution and incubated in the dark at room temperature for 20 min. An enzyme marker was used to measure fluorescence intensities at the excitation and emission wavelengths of 390 nm and 470 nm. A primary function was fitted with WGLU concentration and fluorescence intensity, with H_0_ calculated as the slope of the resulting curve [15].

#### 2.5.5. Measurement of Solubility 

WGLU solution (0.01 g/mL) was prepared in 0.1 mol/L PBS (pH 7.4) and centrifuged at 8000 rpm for 10 min to separate the supernatant. The WGLU content in the supernatant was determined by the Coomassie brilliant blue method. WGLU solubility (%) was calculated according to Equation (2) [16].

(2) $$\mathrm{Solubility}\;(\%)=\frac{\mathrm{Weigh}\;\mathrm{of}\;\mathrm{protein}\;\mathrm{in}\;\mathrm{the}\;\mathrm{supernatant}}{\mathrm{Weigh}\;\mathrm{of}\;\mathrm{total}\;\mathrm{protein}}\times 100$$

#### 2.5.6. Measurement of Water-Holding Capacity (WHC) and Oil-Holding Capacity (OHC)

In a centrifuge tube, 0.5 g of WGLU was combined with 20 mL of distilled water and allowed to hydrate at 27 °C for 2 h. Subsequently, the mixture was centrifuged at 8000 rpm for 10 min, and after the removal of the supernatant the weight of the centrifuge tube containing the sediment was measured [17]. WHC was calculated according to Equation (3). OHC was measured using a similar method, where soybean oil replaced distilled water. Equation (4) was used to compute the OHC value.

(3) $$\mathrm{WHC}\;(g/g)=\frac{W_{3}-W_{1}-W_{2}}{W_{1}}$$

(4) $$\;\mathrm{OHC}\;(g/g)=\frac{W_{3}^{\prime }-W_{1}-W_{2}}{W_{1}}$$

where *W*_1_ is the weight of the WGLU (5 g), *W*_2_ is the weight of the centrifuge tube (g), and *W*_3_ and *W*_3_′ indicate the mass of the centrifuge tube and the sediment that remains (g).

#### 2.5.7. Measurement of Emulsifying Properties

A WGLU solution with a concentration of 0.01 g/mL was made using a 0.1 mol/L PBS solution at pH 7.4, and soybean (5 mL) oil was added to the WGLU solution (15 mL) and homogenized at 10,000 rpm for 5 min. A 0.05 mL portion of the emulsion was drawn from the bottom and mixed with a 0.1% sodium dodecyl sulfate solution. The absorbance at a 500-nm wavelength was measured using an ultraviolet spectrophotometer (UV-2600, Shimadzu, Kyoto, Japan). After 10 min, 0.05 mL of the lower emulsion was sampled again, combined with sodium dodecyl sulfate solution, and its absorbance was recorded. The emulsifying properties were calculated according to Equations (5) and (6) [18].

(5) $${\mathrm{EAI}\;(m}^{2}/g)=2\times 2.303\frac{A_{0}\times N}{C\times \varphi \times 10000}$$

(6) $$\mathrm{ESI}\;(\min)=\frac{A_{0}}{A_{0}-A_{10}}\times 10$$

where EAI is the emulsifying activity index, *N* is the WGLU dilution coefficient (100), *C* is the WGLU concentration (0.02 g/mL), *φ* is the oil volume fraction (0.25), ESI is the emulsion stability index, and *A*_0_ and *A*_10_ represent absorbances of the emulsions at 0 and 10 min.

#### 2.5.8. Measurement of Foaming Properties 

WGLU solution (0.01 g/mL) was prepared in 0.1 mol/L PBS (pH 7.4), and 10 mL of WGLU solution was taken and dispersed at 8000 rpm for 2 min, then quickly transferred to a measuring cylinder. The Foaming properties were calculated according to Equations (7) and (8) [19].

(7) $$\mathrm{FC}\;(\%)=\frac{V_{0}}{V}\times 100$$

(8) $$\mathrm{FS}\;(\%)=\frac{V_{t}}{V_{0}}\times 100$$

where FC (%) is the foaming capacity, FS (%) is foam stability, *V* represents the WGLU solution’s volume (mL), and *V*_0_ and *V_t_* are the foam volumes at 0 and 10 min (mL).

### 2.6. Statistical Analysis

Each experiment was conducted three times, and SPSS version 26.0 was used for data analysis, with significant differences in results indicated at *p* < 0.05, and plotted using Origin 2021.

## 3. Results

### 3.1. Structural Characterization

#### 3.1.1. Fluorescence Spectra Analysis

**Figure 2:**
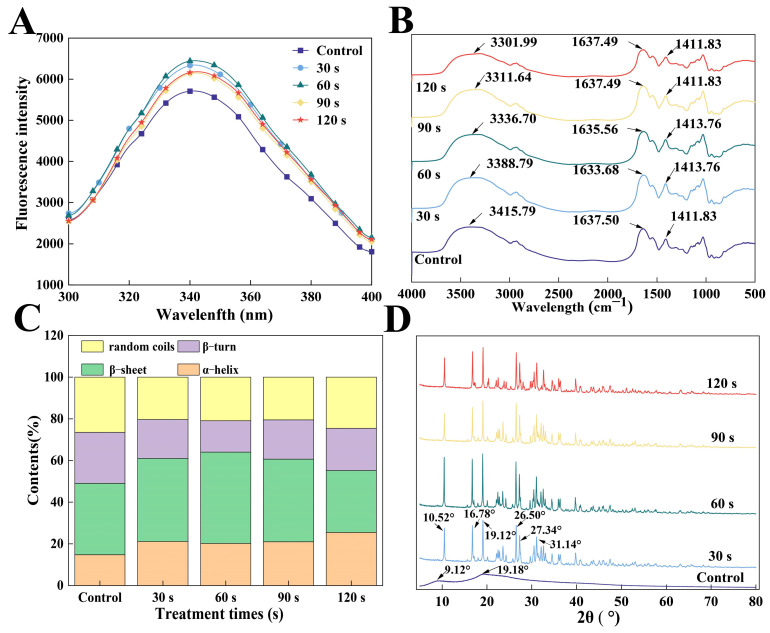
Fluorescence spectra (**A**), Fourier transform infrared spectroscopy (FTIR) spectra (**B**), secondary structure content (**C**), and X-ray diffraction (XRD) spectra (**D**) of WGLU treated with ACP at different times. WGLU, walnut glutenin; ACP, atmospheric cold plasma.

A protein’s fluorescence spectral properties can be used to characterize changes in its tertiary structure. In Figure 2A, it is shown that the control group has a maximum emission wavelength (λmax) of 341 nm, and that of WGLU after ACP treatment showed a slight red shift (λmax = 342 nm). The reason is that WGLU is extracted by strong alkaline solutions, which may damage its structure, and the oxidation effect is enhanced after ACP treatment, resulting in tryptophan residues shifting toward a more polar hydrophilic environment [12,20]. Furthermore, compared to the control group, ACP treatment caused a rise in WGLU fluorescence intensity and showed an increase followed by a decrease. These discrepancies may be related to the fact that ACP treatment produces an etching effect that depolymerizes glutamine in WGLU, exposing the chromophores and enhancing fluorescence intensity [21]. It was observed that the WGLU fluorescence intensity was not the highest at 30 s of ACP treatment, which may be due to the short duration of ACP treatment and incomplete exposure of the chromogenic groups within the protein. ACP treatment for 60 s, the spatial structure of WGLU becomes looser, resulting in maximum fluorescence intensity. When the treatment time reaches 90 s and 120 s, the fluorescence intensity of WGLU decreases. This may be related to the intense oxidation of WGLU during prolonged treatment, leading to carbonyl group formation, aggregation of proteins, and concealment of amino acid residues, leading to a reduction in fluorescence [22].

#### 3.1.2. Fourier Transform Infrared Spectroscopy Analysis

The effects of ACP treatment on the chemical composition and conformation of WGLU were analyzed using FTIR spectroscopy. Figure 2B indicates that the control group exhibits a pronounced characteristic peak within the 3415.79 cm^−1^ band, mainly due to the vibrations from N-H and O-H stretching. After ACP treatment, the absorption peak of WGLU in the 3415.79 cm^−1^ band shifted to lower wavelengths compared to the control group, which indicated that ACP treatment disrupted the hydrogen bonding of WGLU. This disruption can improve the hydrophilic property of the protein by inducing polymer chains or specific functional groups to covalently attach to the protein surface [23]. At 1637.50 cm^−1^, the control group showed a unique absorption peak, corresponding to C-O bond stretching in the protein amide I band, at 1411.83 cm^−1^, the peak largely caused by N-H bending and C-N stretching within the amide II band [24]. After ACP treatment, the absorption peak of WGLU at 1637.50 cm^−1^ showed a blue shift, with the greatest shift occurring at 30 s and 60 s. The result indicated that ACP treatment induced dissociation of the WGLU structure, increasing the electron cloud density of C-O, which caused the blue shift in the absorption peak [13]. At 90 s and 120 s, the absorption peak shifted blue by only 0.01 cm^−1^. This may be due to prolonged processing, causing the cross-linking and aggregation of WGLU structures. Additionally, compared with the control group, the absorption peak of WGLU at 1411.83 cm^−1^ showed a red shift at 30 s and 60 s after ACP modification. High-energy ions and particles may collide with proteins, thereby transferring energy to intermolecular bonds, leading to further unfolding of the WGLU structure [18].

According to Figure 2C, the control group’s secondary structure was primarily β-turn and β-sheet. The β-sheet content of WGLU initially went up and then declined as ACP treatment time was extended, while the α-helix content first decreased and then increased. It has been reported that an increase in α-helix and β-turn indicates protein cross-linking to form macromolecular aggregates, causing peptide chains to contract and become more tightly ordered [13]. A rise in β-sheets and random coil is seen as a sign of increased flexibility, structural disorder, and unfolding of protein [25]. The secondary structure of proteins is dynamically balanced. α-helices, β-folds, β-turns, and random curls together form the local conformation of the protein backbone. They can be interconverted with each other, thus affecting the protein’s structural conformation. The control group had a β-sheet content of 46.62%, an α-helix content of 14.03%, a random coils content of 13.24%, and a β-turn content of 26.11% (Table 1). The secondary structure of the WGLUs was altered after ACP treatment, with the β-sheet content peaking at 60 s of ACP treatment (47.64%), while the α-helix content decreased to 13.81%. Attributed to ACP oxidation of side-chain amino acids destroys the groups responsible for maintaining the ordered spatial structure, resulting in the loss of non-covalent bonds that form the basic secondary structural units [9,26]. After 120 s of ACP treatment, β-sheet and β-turn were transformed into an α-helix, which may cause WGLU re-aggregation and increased protein ordering by prolonged ACP treatment.

#### 3.1.3. X-Ray Diffraction Analysis

In Figure 2D, the control group is shown to have diffraction peaks at 2θ = 9.12° and 19.18°, which are indicative of an amorphous or semi-crystalline structure [27]. After ACP treatment, WGLU exhibited more diffraction peaks at 10.52°, 16.78°, 19.12°, 26.50°, 27.34°, and 31.14°, and these peaks were sharper, indicating an enhanced crystalline structure. This may be because ACP treatment destroys the chemical bonds of WGLU, weakening the regularity of the crystal arrangement and leading to drastic changes in the spatial conformation [28]. It is worth noting that, after ACP treatment, the crystallinity of WGLU increased compared to the control group (38.16%), with values of 78.51%, 91.57%, 79.79%, and 77.26%, respectively. The crystallinity reached the maximum at 60 s, probably due to the oxidative effect produced by ACP, which made the protein structure looser. The crystallinity decreased at 90 s and 120 s. However, due to the prolongation of the ACP treatment time, the disorder of the crystal arrangement of WGLU decreased [29].

#### 3.1.4. Scanning Electron Microscope Analysis

**Figure 3:**
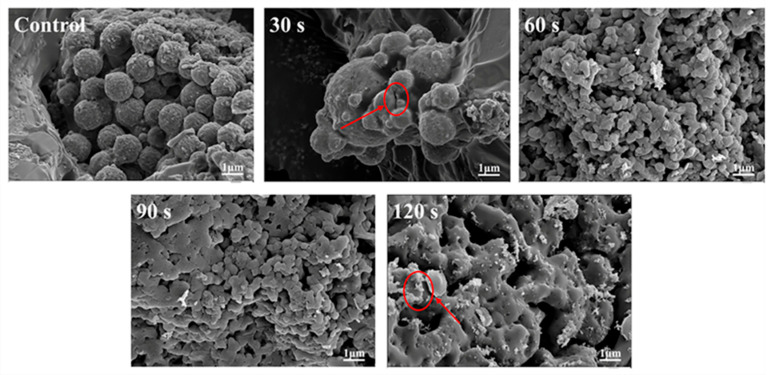
Scanning electron microscopy (SEM) of WGLU after ACP treatment at different times. WGLU, walnut glutenin; ACP, atmospheric cold plasma. The red arrows/circles in the figure indicate significant changes in WalPI after ACP treatment.

Figure 3 shows that the microstructure of the control group displays a rigid spherical structure. After ACP treatment, the closed structure of WGLU was disrupted, presenting an irregular shape with cracks appearing on the surface. Meanwhile, the size of the particles first reduces and then grows as the processing time extends. This effect is caused by the high-energy particles generated by ACP that interact with the surface of WGLU, reducing the size of large particles and significantly modifying their external appearance [30]. At 60 s of ACP treatment, WGLU was mostly fragmented into small particles with the smallest particle size and uniform distribution. This could be because a longer ACP treatment time generates more active substances that bombard the WGLU surface, resulting in a stronger etching effect and creating cavities within the protein [18]. Active substances can also diffuse from these cavities into WGLU, disrupting intermolecular hydrogen bonds and leading to WGLU structural unfolding [31]. However, at 90 s and 120 s of ACP treatment time, WGLU formed aggregates, mostly lumps. This may result from the prolonged treatment period, which strengthens inter-protein interactions and increases the chance of protein-protein binding. Also, when ACP treatment was prolonged to 120 s, the WGLU surface exhibited numerous small fragments and voids. The cause is the presence of a significant quantity of electrons, ions, and other active substances that attacked the surface of WGLU for a long time, generating many low fragments, which could not be aggregated in time, thus leading to their adhesion to the protein surface [32].

### 3.2. Physical and Chemical Properties

#### 3.2.1. Particle Size and Zeta Potential Analysis

**Figure 4:**
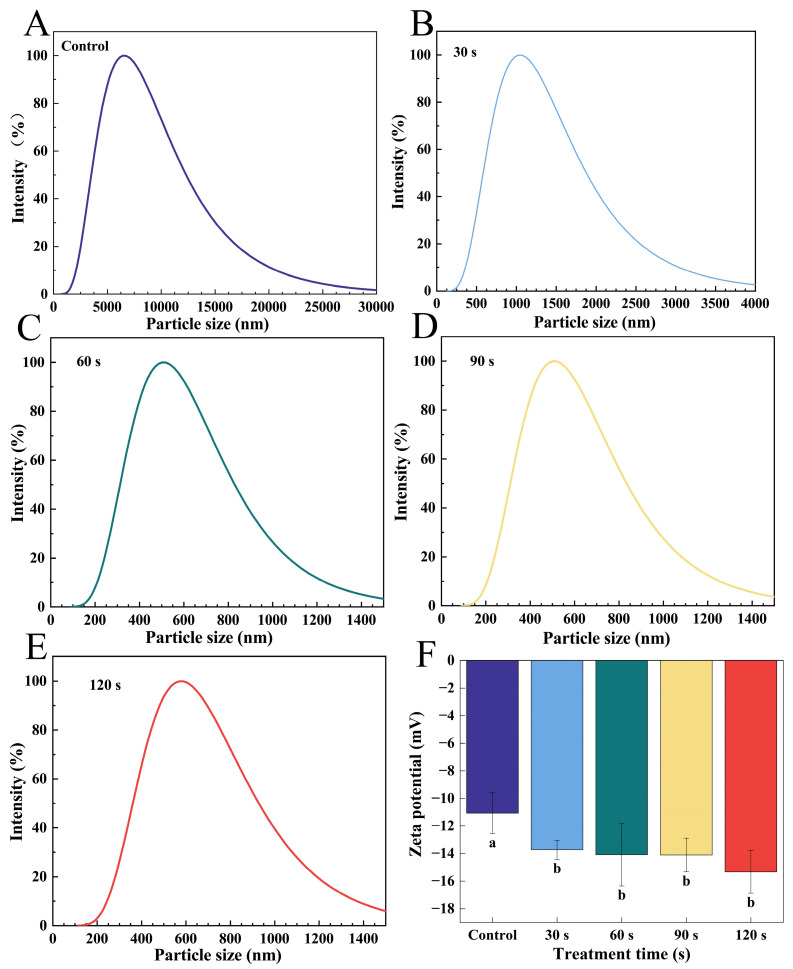
Particle size distribution (**A**–**E**) and zeta potential (**F**) of WGLU treated with ACP at different times. Different lowercase letters (a,b) indicate significant differences between different treatment conditions (*p* < 0.05). WGLU, walnut glutenin; ACP, atmospheric cold plasma.

Figure 4A–E shows the particle size distribution of WGLU with different times of ACP treatment, where the protein particle size distribution was gradually shifted to the left toward smaller particle sizes after ACP treatment compared to the control. This change suggests that ACP treatment may have contributed to the depolymerization or dispersion of protein particles, resulting in the overall particle size reduction. In addition, the peak widths of the particle size distributions tended to decrease and then increase with time, and the peak widths were the smallest at 60 s. The decrease in peak width may mean that the ACP treatment resulted in a more uniform distribution of protein particles at the initial stage, while the subsequent increase in peak width may indicate that the protein particles were further depolymerized or partially aggregated with the extension of the treatment time, resulting in a wider range of particle size distribution [33]. As shown in Table 2, the average particle size of the control group was 6201.45 nm, and the average particle size of WGLU decreased after ACP treatment and exhibited a trend of first declining and then rising as treatment time increased. This occurred primarily because electrons were released, ions, and neutral particles during ACP discharge, which eroded the protein surface and disrupted the apparent densities of thin layers, leading to a reduction of the large particles [34]. The smallest average particle size of 341.70 nm for WGLU was recorded at 60 s of ACP treatment. The cause could be the elevated density of charged particles and ions resulting from ACP, which disrupts the molecular forces between proteins and reduces WGLU particle aggregation [18]. WGLU’s particle size averaged 543.08 nm and 573.48 nm at 90 s and 120 s of ACP treatment. This increase is due to the large amount of reactive chemicals, such as O_3_ and H_2_O_2_, which can oxidize amino acid side chains, especially cysteine, promoting protein–protein binding. Furthermore, the binding of polar compounds to protein surfaces can increase surface energy and adhesive strength [35]. The surface potential of a protein in solution is represented by its zeta potential. Usually, the physical stability of proteins in an aqueous solution is enhanced by a rise in the absolute zeta potential value [36].

Figure 4F shows that the control group’s zeta potential was −11.06 mV, and the zeta potentials of WGLU increased after ACP treatment, initially rising and then falling as ACP treatment time increased. This was largely because ACP treatment led to the creation of active particles like N^2+^, O_3_, and H_2_O_2_, which led to the breakdown of soluble aggregates, revealing additional polar groups on the WGLU surface and enhancing the zeta potential [11]. At 60 s of ACP treatment, zeta potential reached its maximum (−15.33 mV), attributed to the high density of chemical reactants generated by ACP, which increased the oxidation of amino acid residues and converted them into negatively charged byproducts [22]. Furthermore, ACP treatment can lead to the degradation of WGLU aggregates or conformational changes in secondary structures, resulting in the exposure of some previously buried amino acids and an increase in zeta potential. At 90 s and 120 s of ACP treatment, the zeta potential of WGLU was −14.10 and −14.08 mV, which may be due to WGLU aggregation and reduced exposure of polar groups, leading to a decrease in zeta potential.

#### 3.2.2. Total and Active -SH Analysis

**Figure 5:**
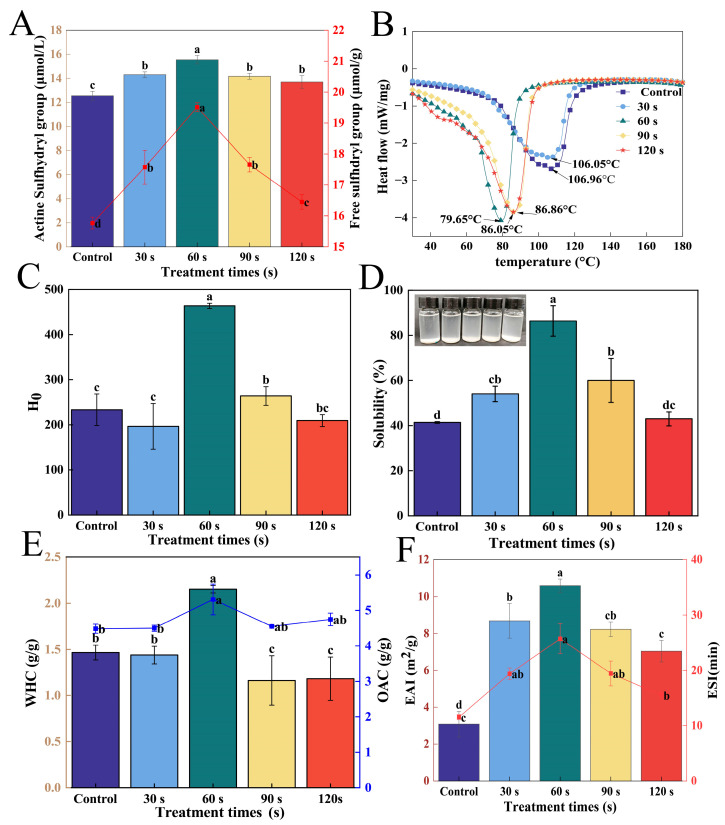
Total and active sulfhydryl (**A**), thermal stability (DSC) (**B**), surface hydrophobicity (H_0_) (**C**), solubility (**D**), water-holding capacity (WHC), oil-holding capacity (OHC) (**E**), and emulsification activity index (EAI) and emulsification stability index (ESI) (**F**) of WGLU treated with ACP at different times. Different lowercase letters (a–d) indicate significant differences between various treatment conditions (*p* < 0.05). WGLU, walnut glutenin; ACP, atmospheric cold plasma.

Alterations in free -SH groups can influence the functional characteristics of proteins. In Figure 5A, it is shown that the active -SH group of the control group was 12.55 μmol/g and the total -SH group was 15.77 μmol/g. However, following treatment with ACP, WGLU showed a significant increase (*p* <0.05) in its active and total -SH groups and displayed an initial rise followed by a decline with longer treatment durations. It may result from the tertiary structure unfolding and the disulfide bonds between amino acid side chains breaking [37]. Meanwhile, active -SH groups were positively correlated with total -SH groups. WGLU’s active and total -SH groups peaked at 60 s during ACP treatment, showing an increase of 23.90% and 23.72% compared to the control group. This increase is primarily attributed to the disaggregation of WGLU aggregates and the reduction in particle size, which makes the -SH groups more accessible to react with the active substances generated by ACP [22]. It is noteworthy that prolonged treatment may lead to increased oxidation, prompting the reaggregation of WGLU and an increase in disulfide bonds, thereby decreasing the amount of free -SH groups.

#### 3.2.3. Thermal Stability Analysis

The thermal stability of WGLU was evaluated using DSC, and its thermal stability was related to the hydrogen bond content within the protein structure [38]. As shown in Figure 5B, all WGLU exhibited a single broad endothermic peak, which was primarily attributed to the denaturation of the protein’s tertiary or quaternary structure. The peak denaturation temperature (Td) reflects the thermal stability of proteins, with higher Td indicating denser protein structures and more stable configurations [39]. In the control group, Td was 106.96 °C. After ACP treatment, WGLU’s Td decreased, exhibiting a trend of initially decreasing followed by an increase as the ACP treatment time extends. This could be a result of ACP’s high-energy particles impacting WGLU’s surface structure, breaking its hydrogen bonds and weakening intermolecular forces, resulting in the WGLU structure transitioning from a dense state to a loose state [28]. The Td of WGLU was the lowest at 60 s of ACP treatment (79.65 °C). This may be ACP-induced oxidation, which increases electrostatic repulsion and steric hindrance between protein molecules, leading to a more open WGLU structure and reduced particle size, thereby reducing its ability to withstand higher temperatures. The Td of WGLU increased at 90 and 120 s of ACP treatment, which may be due to the prolonged time of ACP treatment, which causes WGLU to cross-link oxidatively and form aggregates.

#### 3.2.4. Surface Hydrophobicity Analysis

Differences in aggregation and folding of protein tertiary structures can be assessed using H_0_ [40]. Figure 5C illustrates that the control group’s H_0_ was measured at 233.43. Following ACP treatment, the H_0_ of WGLU first rose and then fell as the duration of treatment grew. The H_0_ of WGLU was lower than that of the control group at 30 s of ACP treatment, probably because the energetic particles generated by ACP rapidly broke the C-C and C-H bonds on the surface of the material and caused them to react with reactive oxygen species in the plasma environment or oxygen in the treated air. The introduction of polar functional groups in WGLU led to its rapid oxidation, resulting in a significant decrease in H_0_ (*p* < 0.05) [22]. The H_0_ of WGLU was all greater than that of the control group with the increase in ACP treatment time, which may be due to the ACP etching effect leading to an increase in the roughness of the protein surface or the dissociation of the protein subunits, which leads to the observed increase in H_0_ [41]. The H_0_ of WGLU peaked at 60 s of ACP treatment (463.50), an increase of 98.56% compared to the control group. The rise is attributed to the adequate processing time changing the spatial structure of WGLU, which causes the WGLU structure to unfold and exposes hydrophobic amino acids, increasing the polarity of the water environment, and enabling ANS to fully bind with hydrophobic amino acids [42]. Li et al. [43] studied the effects of various modification methods on walnut protein and found that the hydrophobicity of the walnut protein surface decreased after ACP modification. This decrease is likely due to ACP-induced oxidation and cross-linking, which increase the oxygen-containing groups on walnut protein, expose polar groups, and reduce the hydrophobic regions. At 90 s and 120 s of ACP treatment, the H_0_ of WGLU were 263.77 and 209.20, exhibiting a notable downward trend (*p* < 0.05). The decrease in hydrophobicity is due to the aggregation of WGLUs by electrostatic attraction, and the hydrophobic sites bound by ANS probes may be covered, resulting in a decrease in hydrophobicity.

#### 3.2.5. Solubility Analysis

Protein solubility can evaluate the interaction between amino acid residues on the protein surface and water, as well as protein-protein interactions [42]. In Figure 5D, it is shown that the control group had a solubility of 41.38%. The solubility of WGLU increased significantly after ACP treatment (*p* < 0.05) and showed an upward trend initially, followed by a downward trend as ACP treatment time progressed. This increase was primarily attributed to the high-energy active particles generated by ACP, which disrupted the spatial structure of WGLU. This disruption caused protein collisions, reduced the particle size, and increased the specific surface area and the number of charged groups, thereby strengthening the interaction between WGLU and water and leading to better solubility [44]. The WGLU solubility reached the maximum at 60 s of ACP treatment (86.35%). It may be that ACP produced more energetic electrons, ions, and a significant amount of free radicals, which increase the surface energy of the hydration layer on the protein surface, speeding up the movement of water and facilitating the binding of WGLU particles to water [45]. Additionally, the unfolding of the WGLU structure, reduction in size, increase in surface potential, and increase in H_0_ all contributed to enhanced solubility. The WGLU solubility was significantly reduced (*p* < 0.05) at 90 s and 120 s of ACP treatment. Prolonged treatment likely exposes too many active sites, causing WGLU to aggregate and reducing its interaction with water.

#### 3.2.6. Water and Oil Holding Capacity

The ability of proteins to gel, emulsify, and foam is largely dependent on WHC and OHC, which affect their interactions with water and oil, respectively. Figure 5E shows that the WHC of the control group was 1.47 g/g, while the OHC was 4.49 g/g. After ACP treatment, the WHC and OHC of WGLU initially increased and then decreased, showing a clear trend concerning the treatment time. WGLU exhibited a WHC of 2.15 g/g and an OHC of 5.31 g/g, at 60 s of ACP treatment, which were increased by 46.26% and 18.26% compared to the control group. This is attributed to the unfolding of the tertiary structure of WGLU, which increases hydrophobicity and allows the protein to absorb more water and oil [46]. In addition, this may also be related to the increase in protein zeta potential. The WHC and OHC of WGLU decreased at 90 and 120 s of ACP treatment due to protein aggregation and the reduction of hydrophobic sites available for binding water and oil. The reduction in WHC might result from the decreased solubility of WGLU and the creation of protein aggregates. The decrease in OHC may be due to the reduced hydrophobicity and increased hydrophilicity of the protein surface with prolonged ACP treatment. Since OHC is positively correlated with H_0_, the decrease in hydrophobicity likely leads to reduced OHC [47].

#### 3.2.7. Emulsification Activity Index and Emulsion Stability Index Analysis

EAI and ESI can assess the ability of proteins to form oil-water interfaces and stabilize emulsion droplets [48]. As shown in Figure 5F, the control group’s EAI was 3.09 m^2^/g, and its ESI was 11.54 min. After ACP treatment, both EAI and ESI of WGLU exhibited notable increases (*p* < 0.05) and displayed a trend of increasing before decreasing as the duration of ACP treatment extended. The improvement in emulsification performance is the result of significant increases in WGLU zeta potential, H_0_, and OHC. These changes promoted the dispersion and adsorption of protein at the water-oil interface [49]. The WGLU’s EAI and ESI achieved their peak values at 60 s of ACP treatment, which were increased by 2.43 and 1.23 times compared to the control group. The reason for this is the increased solubility of WGLU, which facilitates its migration to the water-oil interface. Nevertheless, the EAI and ESI of the WGLU significantly decreased (*p* < 0.05) at 90 s and 120 s of ACP treatment. This suggests that the prolonged ACP treatment time resulted in the formation of covalent cross-links within or between proteins. Consequently, the interaction between the protein and small oil droplets is hindered, leading to a reduction in the emulsifying capacity of WGLU [50].

#### 3.2.8. Foaming and Foam Stability Analysis

**Figure 6:**
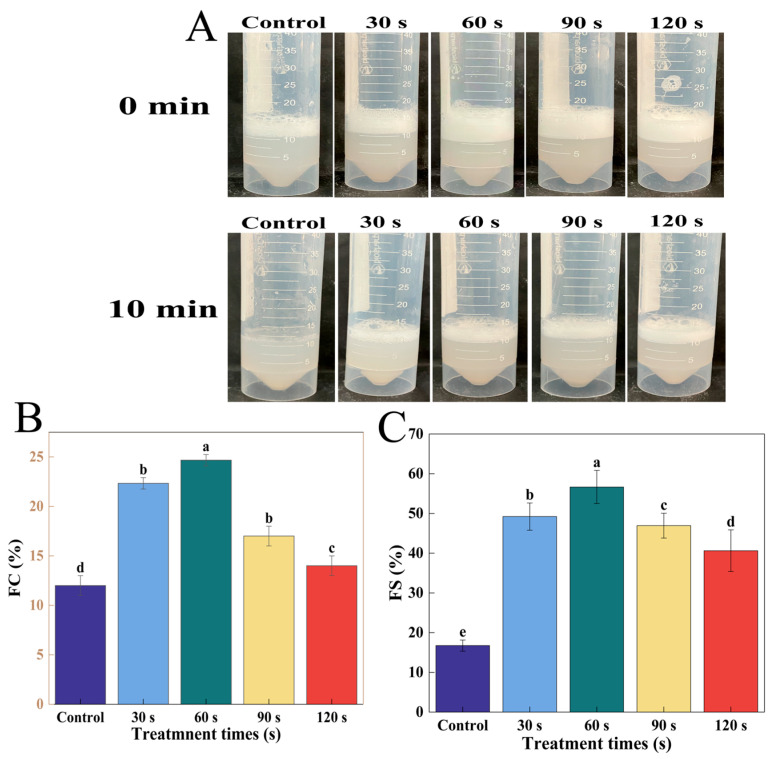
Foaming visualization diagram (**A**) and foaming capacity (FC) (**B**) and foam stability (FS) (**C**) of WGLU treated with ACP at different times. Different lowercase letters (a–e) indicate significant differences between different treatment conditions (*p* < 0.05). WGLU, walnut glutenin; ACP, atmospheric cold plasma.

Proteins are amphiphilic and can stabilize air–water (foam) interfaces by forming a thin, flexible, and stable interfacial film that reduces surface tension, facilitating foam formation and stabilization [43]. Figure 6A is the foam visual diagram of WGLU, which can help more intuitively understand the foam characteristics between different samples. The foam in the control group had nearly dissipated after 10 min, and the stability of WGLU was poor. Figure 6B,C illustrates that the control group exhibited an FC of 12% and an FS of 16.74%. In addition, both the FC and FS of WGLU increased after ACP treatment and displayed a tendency to rise initially and then decrease with prolonged ACP treatment time. This occurs because the protein side chains unfold, resulting in a more flexible structure and exposing more hydrophobic sites, which facilitates adsorption at the air–water interface, resulting in more foam [18]. The increased FC is associated with enhanced H_0_, as the exposure of hydrophobic residues strengthens protein–air interactions, thereby promoting foam formation [51]. ACP treatment has been shown to cause oxidation in the side chains of amino acids, thus enhancing the flexibility of proteins and improving their adsorption rate at the air–water interface [52]. At 60 s of ACP treatment, the FC of WGLU was 24.67%, likely as a result of the WGLU secondary structure unraveling, which enhances hydration and protein–water interactions, thereby maximizing FC. The FS of WGLU reached a maximum (56.67%) at 60 s, which was attributed to the increase in H_0_, enhanced structural integrity of the interfacial membrane, and the formation of a protein network that acts as a mechanical barrier to bubble breakage and aggregation, thus improving the foam stability of the WGLU [2]. The FC and FS of WGLU decreased at 90 s and 120 s of ACP treatment, likely due to the formation of aggregates, a reduction in hydrophobicity, and an increase in particle size, which hindered the protein’s ability to adsorb efficiently at the air–water interface. Extended treatment resulted in reduced solubility of WGLU, which further hindered its adsorption and negatively affected foam formation and stability.

## 4. Conclusions

The ACP treatment effectively disrupted the spatial structure of WGLU, disrupting its secondary structure and unfolding its tertiary structure, thereby significantly enhancing surface hydrophobicity. Secondly, the duration of ACP treatment is closely linked to the structural alterations and functional characteristics of WGLU. At 60 s into ACP treatment, WGLU reached its smallest particle size and greatest zeta potential, enhancing its physicochemical properties, notably solubility and emulsifying activity. However, prolonged treatment resulted in the formation of WGLU aggregates, and, in particular, their WHC and FS were significantly reduced. Overall, ACP treatment for the appropriate time, especially 60 s, represents an effective strategy to improve the structural and functional characteristics of WGLU. This study can provide valuable insights for the future development of plant-based food. However, further studies on the long-term stability and oxidation resistance of ACP-treated WGLUs are needed. Follow-up studies could use molecular dynamics to investigate the effect of ACP treatment on the structure of WGLU.

## Figures and Tables

**Table 1:** Secondary structure content of WGLU after ACP treatment at different times.

Treatment Times (s)	β-Sheet (%)	Random Coil Content (%)	α-Helix (%)	β-Turn (%)
Control	46.62	13.24	14.03	26.11
30 s	45.57	13.07	13.98	27.38
60 s	47.64	13.21	13.81	25.34
90 s	46.94	12.98	13.80	26.28
120 s	44.98	13.19	28.28	13.55

**Table 2:** Average particle size of WGLU after atmospheric cold plasma treatment at different times.

Treatment Times (s)	Average Particle Size (nm)
Control	6201.45 ± 98.63 ^a^
30 s	960.00 ± 84.27 ^b^
60 s	341.70 ± 6.95 ^c^
90 s	543.08 ± 29.85 ^d^
120 s	573.66 ± 4.77 ^c^

Different lowercase letters (^a^–^d^) indicate significant differences between various treatment conditions (*p* < 0.05).

## Data Availability

The original contributions presented in this study are included in the article. Further inquiries can be directed to the corresponding authors.
